# An integrative model for the comprehensive classification of *BRCA1* and *BRCA2* variants of uncertain clinical significance

**DOI:** 10.1038/s41525-022-00302-3

**Published:** 2022-06-03

**Authors:** Edwin S. Iversen, Gary Lipton, Steven N. Hart, Kun Y. Lee, Chunling Hu, Eric C. Polley, Tina Pesaran, Amal Yussuf, Holly LaDuca, Elizabeth Chao, Rachid Karam, David E. Goldgar, Fergus J. Couch, Alvaro N. A. Monteiro

**Affiliations:** 1 Department of Statistical Science, Duke University, Durham, NC 27708 USA; 2 Department of Health Sciences Research, Mayo Clinic, Rochester, MN 55901 USA; 3 Department of Laboratory Medicine and Pathology, Mayo Clinic, Rochester, MN 55902 USA; 4 Ambry Genetics Corporation, Aliso Viejo, CA 92656 USA; 5 Department of Dermatology, University of Utah School of Medicine, Salt Lake City, UT 84132 USA; 6 Cancer Epidemiology Program, H. Lee Moffitt Cancer Center & Research Institute, Tampa, FL 33612 USA

**Keywords:** Molecular medicine, Cancer genetics, Breast cancer

## Abstract

Loss-of-function variants in the *BRCA1* and *BRCA2* susceptibility genes predispose carriers to breast and/or ovarian cancer. The use of germline testing panels containing these genes has grown dramatically, but the interpretation of the results has been complicated by the identification of many sequence variants of undefined cancer relevance, termed “Variants of Uncertain Significance (VUS).” We have developed functional assays and a statistical model called VarCall for classifying *BRCA1* and *BRCA2* VUS. Here we describe a multifactorial extension of VarCall, called VarCall XT, that allows for co–analysis of multiple forms of genetic evidence. We evaluated the accuracy of models defined by the combinations of functional, in silico protein predictors, and family data for VUS classification. VarCall XT classified variants of known pathogenicity status with high sensitivity and specificity, with the functional assays contributing the greatest predictive power. This approach could be used to identify more patients that would benefit from personalized cancer risk assessment and management.

## Introduction

The recent widespread utilization of clinical DNA sequencing has facilitated the implementation of precision medicine where clinical decisions, treatments, and procedures are tailored to each patient based on the identification of DNA variants. However, while a genetic variant may be identified in individuals with the disease, the likelihood of pathogenicity of the variant may not be sufficient to justify an intervention. Thus, genetic variants of uncertain clinical significance (VUS) present a critical challenge to the effective use of clinical genetic information^1^.

*BRCA1* and *BRCA2* are among the genes most frequently subjected to clinical genetic testing because of the clinical importance of pathogenic variants. Carriers of pathogenic variants in *BRCA1* or *BRCA2* are at significantly increased risk (Relative Risk (RR) > 4) for breast and ovarian cancer and this knowledge can guide screening for early detection and the use of risk-reducing prophylactic surgeries. Importantly, the discovery of pathogenic variants also identifies patients likely to benefit from PARP inhibitors, a class of therapeutic agents that target cancers with homologous recombination DNA repair deficiency. Thus, the determination of the clinical relevance of VUS in *BRCA1* and *BRCA2* has significant implications for clinical care.

Currently, several non-mutually exclusive approaches exist for classifying *BRCA1* and *BRCA2* VUS. For example, loss-of-function variants in these tumor suppressors, for which the effect on protein function can be inferred from the genetic code (frameshift, nonsense, or consensus splicing acceptor/donor sites), are generally classified as pathogenic. A multifactorial model that includes family history, co-segregation, and co-occurrence with another known pathogenic variant in the same gene (biallelic inactivation is embryonic lethal and in the case of *BRCA2* can cause Fanconi anemia) has been used for the remaining variant types (inframe indels, synonymous and missense in coding changes; intronic and regulatory)^2,3^. It is also possible to classify variants based on the ACMG/AMP (The American College of Medical Genetics and Genomics; Association for Molecular Pathology) rule-based guidelines, in which pathogenicity may be predicted using multiple sources of the available evidence, including results from reproducible and clinically validated functional assays^4,5^. However, current *BRCA1* and *BRCA2* variant classification models either do not incorporate functional data or use these data-only as supporting evidence, for or against pathogenicity^4–6^. The potential for the application of functional data has not been fully realized because there is not an adequate statistical framework with which to (a) optimize the discrimination between pathogenic and non-pathogenic variants using functional data; and (b) integrate functional data with family history and other sources of data to obtain a global likelihood of pathogenicity.

Here, we present VarCall XT (VarCall eXTended), a Bayesian statistical framework that incorporates data from established functional assays with family data and sequence-based in silico prediction models. This model was used to determine the likelihood of pathogenicity for individual missense variants in *BRCA1* and *BRCA2* in the context of hereditary breast and ovarian cancer syndrome (HBOC), and to examine the effect on predictive accuracy realized by adding the available family or in silico data to the functional data.

## Results

### Comparative results—classification accuracy

**Fig. 1:**
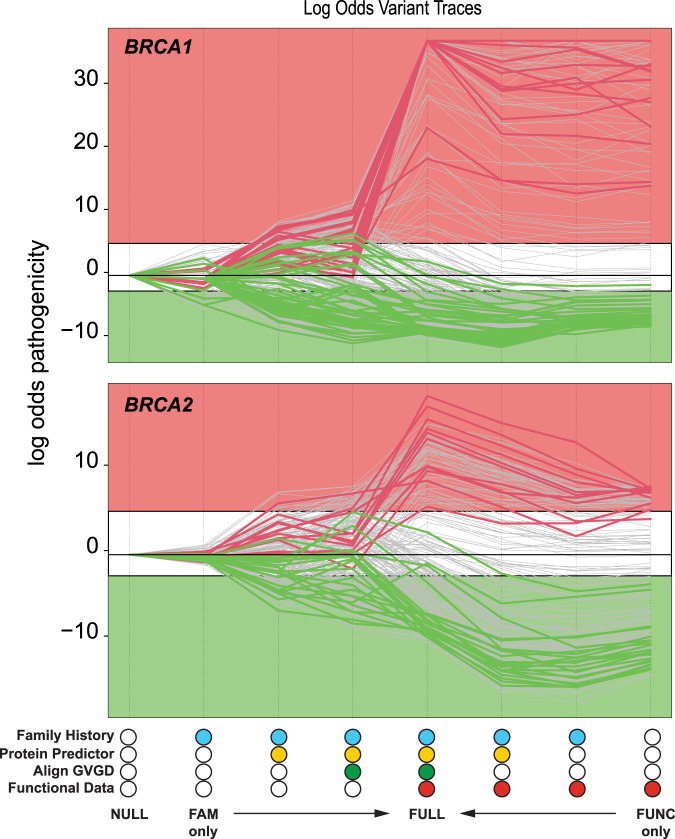
Trace plots of individual variant log-odds (*y*-axis) of pathogenicity across models (*x*-axis) for *BRCA1* (top) and *BRCA2* (bottom). The legend indicates the data elements included in the model with filled circles; the value plotted for the `Null' model (i.e., the model with no data) is the marginal probability of pathogenicity estimated by the model including all data elements (labelled `Full'). Variants known a priori to be pathogenic are plotted in red, while those known to be benign are plotted in green; the VUS are plotted in gray. The light red region corresponds to posterior probabilities of pathogenicity of 99% or higher (IARC class 5), while the light green region corresponds to posterior probabilities of pathogenicity of 5% or lower (IARC classes 1 and 2); values in the white band fall within the `no-call' range.

VarCall models make a binary prediction of pathogenic versus benign classification for each variant. In Fig. 1, we trace the estimated log-odds of pathogenicity for each variant based on the two progression series for *BRCA1* or *BRCA2* described above. While VarCall analyses of the functional data used here have been previously reported^7,8^, we refit these models using our VarCall XT software and summarize these fits here for the sake of consistency and to provide a basis of comparison for the multifactorial extensions of these models.

We initially considered the progression from the null to full models. Classification probabilities for both genes largely remain in the no-call range (VUS) based on the family data alone (Fig. 1). Only three of 96 variants, all in *BRCA1*, had enough family data for classification (all three were correctly classified as benign; Supplementary Table 1). The addition of the protein predictor data provided increased resolution and a greater degree of separation between the known pathogenic and benign variants, with 45 variants classified as benign or pathogenic (Fig. 1). In all cases, the classifications were correct, but benign variants were twice as likely to reach the threshold of classification as pathogenic variants. With the addition of the Align-GVGD score to the model with family and in silico data, six more variants were classified. However, three *BRCA1* benign variants were incorrectly called pathogenic. The addition of the functional data, resulting in the “full model”, provided the clearest improvement in predictive accuracy, with 89 of the 96 variants correctly classified (Fig. 1).

Next, we took the original function-based VarCall model as the starting point. This model achieved 98% accuracy, with 94 of the 96 variants correctly classified and one *BRCA1* benign and one *BRCA2* pathogenic variant remaining as VUS. Progressively adding the family and protein predictor data diminished overall accuracy with four variants left unclassified. Progressing to the full model with the addition of the Align-GVGD score further diminishes accuracy with seven variants remaining unclassified (Fig. 1).

Table 1 displays operating characteristics estimates for the full set of fifteen predictive models when applied to the *BRCA1* and *BRCA2* data sets and computed using the *n* = 63 *BRCA1* and *n* = 33 *BRCA2* variants of known status. It is clear that the functional assays contributed the greatest predictive power to the VarCall XT model. The models that included the functional data all had sensitivity and specificity estimates greater than 0.65 and frequently exceeded 0.9. Models that did not use functional data were all lower than 0.65, because of the modest predictive power of the protein predictor data, Align-GVGD score, and family data.

**Table 1:** Operating characteristics of the *BRCA1* and *BRCA2* predictive models.

		Benign	False	False	Pathogenic		Brier
BRCA1 model	Specificity	No call rate	Positive rate	Negative rate	No call rate	Sensitivity	Score
Function only (original VarCall model)	95.4 (89.3, 99.9)	3.4 (0, 8.8)	1.1 (0, 4.4)	2.2 (0, 8.5)	2.2 (0, 8.5)	95.6 (87, 100)	0.0011
Function + Prot Pred	93.1 (85.6, 99.2)	5.7 (0.4, 12.6)	1.1 (0, 4.4)	2.2 (0, 8.5)	2.2 (0, 8.5)	95.6 (87, 100)	0.0027
Function + family data	95.4 (89.3, 99.9)	3.4 (0, 8.8)	1.1 (0, 4.4)	2.2 (0, 8.5)	2.2 (0, 8.5)	95.6 (87, 100)	8e-04
Function + AGVGD	95.4 (89.3, 99.9)	3.4 (0, 8.8)	1.1 (0, 4.4)	2.2 (0, 8.5)	2.2 (0, 8.5)	95.6 (87, 100)	0.0114
Function + Prot Pred + family data	93.1 (85.6, 99.2)	5.7 (0.4, 12.6)	1.1 (0, 4.4)	2.2 (0, 8.5)	2.2 (0, 8.5)	95.6 (87, 100)	0.0018
Function + Prot Pred + AGVGD	88.5 (79, 96.9)	10.3 (2.4, 19.4)	1.1 (0, 4.4)	2.2 (0, 8.5)	2.2 (0, 8.5)	95.6 (87, 100)	0.0814
Function + family data + AGVGD	95.4 (89.3, 99.9)	3.4 (0, 8.8)	1.1 (0, 4.4)	2.2 (0, 8.5)	2.2 (0, 8.5)	95.6 (87, 100)	0.0086
Function + Prot Pred + family data + AGVGD	88.5 (79, 96.9)	10.3 (2.4, 19.4)	1.1 (0, 4.4)	2.2 (0, 8.5)	2.2 (0, 8.5)	95.6 (87, 100)	0.0718
Prot Pred + family data + AGVGD	65.5 (51.5, 79.2)	26.4 (14, 39.5)	8 (1.3, 16.1)	2.2 (0, 8.5)	55.6 (35.6, 75.3)	42.2 (22.7, 62.1)	0.5636
Prot Pred + AGVGD	63.2 (49, 77.1)	28.7 (15.9, 42.1)	8 (1.3, 16.1)	2.2 (0, 8.5)	55.6 (35.6, 75.3)	42.2 (22.7, 62.1)	0.5663
Family data + AGVGD	8 (1.3, 16.1)	90.8 (82.3, 98.2)	1.1 (0, 4.4)	6.7 (0, 16.8)	91.1 (79.6, 99.7)	2.2 (0, 8.5)	1.0118
Prot Pred + family data	63.2 (49, 77.1)	35.6 (21.9, 49.8)	1.1 (0, 4.4)	2.2 (0, 8.5)	60 (40.2, 79.3)	37.8 (18.7, 57.4)	0.4602
Prot Pred data only	60.9 (46.5, 75)	35.6 (21.9, 49.8)	3.4 (0, 8.8)	2.2 (0, 8.5)	60 (40.2, 79.3)	37.8 (18.7, 57.4)	0.4614
Family data only	8 (1.3, 16.1)	90.8 (82.3, 98.2)	1.1 (0, 4.4)	2.2 (0, 8.5)	95.6 (87, 100)	2.2 (0, 8.5)	1.2588
AGVGD data only	1.1 (0, 4.4)	97.7 (93.2, 100)	1.1 (0, 4.4)	2.2 (0, 8.5)	95.6 (87, 100)	2.2 (0, 8.5)	1.0399

		Benign	False	False	Pathogenic		Brier
BRCA2 model	Specificity	No call rate	Positive rate	Negative rate	No call rate	Sensitivity	Score
Function only (original VarCall model)	95.7 (87.5, 100)	2.1 (0, 8.1)	2.1 (0, 8.1)	4 (0, 15.1)	12 (0, 29.4)	84 (64.6, 99.4)	2e-04
Function + Prot Pred	91.5 (80.5, 99.8)	6.4 (0, 16.1)	2.1 (0, 8.1)	4 (0, 15.1)	4 (0, 15.1)	92 (77.1, 100)	0.0083
Function + family data	95.7 (87.5, 100)	2.1 (0, 8.1)	2.1 (0, 8.1)	4 (0, 15.1)	28 (6.1, 51.8)	68 (43.2, 91.2)	0.0037
Function + AGVGD	87.2 (74, 98.4)	10.6 (0.8, 22.8)	2.1 (0, 8.1)	4 (0, 15.1)	4 (0, 15.1)	92 (77.1, 100)	0.181
Function + Prot Pred + family data	91.5 (80.5, 99.8)	6.4 (0, 16.1)	2.1 (0, 8.1)	4 (0, 15.1)	12 (0, 29.4)	84 (64.6, 99.4)	8e-04
Function + Prot Pred + AGVGD	78.7 (62.4, 93.6)	19.1 (5, 34.8)	2.1 (0, 8.1)	4 (0, 15.1)	4 (0, 15.1)	92 (77.1, 100)	0.2053
Function + family data + AGVGD	87.2 (74, 98.4)	10.6 (0.8, 22.8)	2.1 (0, 8.1)	4 (0, 15.1)	4 (0, 15.1)	92 (77.1, 100)	0.0049
Function + Prot Pred + family data + AGVGD	83 (68, 96.2)	14.9 (2.6, 29)	2.1 (0, 8.1)	4 (0, 15.1)	4 (0, 15.1)	92 (77.1, 100)	0.1118
Prot Pred + family data + AGVGD	48.9 (29.4, 68.6)	48.9 (29.4, 68.6)	2.1 (0, 8.1)	4 (0, 15.1)	76 (53.4, 96.2)	20 (1.9, 41.2)	0.6078
Prot Pred + AGVGD	44.7 (25.3, 64.3)	48.9 (29.4, 68.6)	6.4 (0, 16.1)	4 (0, 15.1)	76 (53.4, 96.2)	20 (1.9, 41.2)	0.6005
Family data + AGVGD	6.4 (0, 16.1)	91.5 (80.5, 99.8)	2.1 (0, 8.1)	4 (0, 15.1)	92 (77.1, 100)	4 (0, 15.1)	1.1563
Prot Pred + family data	40.4 (21.4, 59.8)	57.4 (37.9, 76.6)	2.1 (0, 8.1)	4 (0, 15.1)	84 (64.6, 99.4)	12 (0, 29.4)	0.2373
Prot Pred data only	36.2 (17.7, 55.2)	61.7 (42.4, 80.4)	2.1 (0, 8.1)	4 (0, 15.1)	76 (53.4, 96.2)	20 (1.9, 41.2)	0.2272
Family data only	2.1 (0, 8.1)	95.7 (87.5, 100)	2.1 (0, 8.1)	4 (0, 15.1)	92 (77.1, 100)	4 (0, 15.1)	1.0582
AGVGD data only	2.1 (0, 8.1)	95.7 (87.5, 100)	2.1 (0, 8.1)	4 (0, 15.1)	92 (77.1, 100)	4 (0, 15.1)	1.1937

Point estimates and 95% high-density credible intervals (in parentheses). All values except the Brier score are expressed as percentages.

The original functional assay-based VarCall model was the top model based on overall accuracy for both genes and, based on the scaled Brier score, was the top *BRCA2* model and the second-ranked *BRCA1* model (Supplementary Table 1). Furthermore, its accuracy was nearly as great when only the positive and negative controls where labeled: the average absolute difference in classification probabilities between the full and reduced training set fits was 0.00091 for the *BRCA1* model and 0.0027 for the *BRCA2* model (see Supplementary Fig. 3 and the discussion in Supplemental Results).

Because resolution to estimate predictive accuracy was limited by the number of variants of known status it was difficult to compare VarCall XT models. However, the contrast between models with and without the functional data was clear. The 95% CI limits for sensitivity among the models that excluded the functional data all fell below the lower 95% CI limits of all models that included functional data, while those for specificity did so in the majority of circumstances. Further, overall accuracy estimates (Supplementary Table 1) of the models with functional data (*BRCA1* minimum = 93.7%, *BRCA2* minimum = 87.9%) were uniformly higher than for those models excluding the functional data (*BRCA1* maximum = 58.7%; *BRCA2* maximum = 39.4%). Finally, Brier scores and the no-call rates were systematically lower among the models with function data than those without. Thus, when considering sensitivity and specificity jointly, the least accurate model with functional data was significantly more accurate than the most accurate model without functional data.

### Predictive contribution of the family data

Family history data were available for 86 (25%) of the *BRCA1* variants and 112 (45%) of the *BRCA2* variants. Among the classified variants there were 17 *BRCA1* (seven benign and 10 pathogenic) and 12 *BRCA2* variants (two benign and 10 pathogenic) with family history summary data. When available, the number of families contributing to these data was often small (see Supplemental Data; Variant-level data tab, columns AT and AU). This highlights the fact that even when testing is widespread and prevalent, there will be no available family data on a large fraction of VUS that arise and the data that are available will often provide modest information overall.

We computed ratios of the estimated odds (ORs) in favor of pathogenicity, given both the function and family data, to the estimated odds given the functional data alone. The estimated mean OR among benign variants across both genes was <1.0 as expected (0.31, 95% CI 0.17–0.57) and was >1.0 among known pathogenic variants (OR 1.04, 95% CI 0.39, 2.81). On average, the effect of adding the family data moved the associated classification probabilities marginally in the correct direction; however, this pattern was clearer and more consistent among the known benign variants than the known pathogenic variants, as shown by the wider interval estimates. Note that the inclusion of the family data changes the posterior odds of pathogenicity of all variants, not just those with the data. Estimated classifications of the variants with those data shift causing estimates of the population (of variants) level parameters that specify the Gaussian classification model to adjust in response, thereby altering the mapping, applicable to all variants, between functional effects estimates and their associated likelihoods of pathogenicity.

**Fig. 2:**
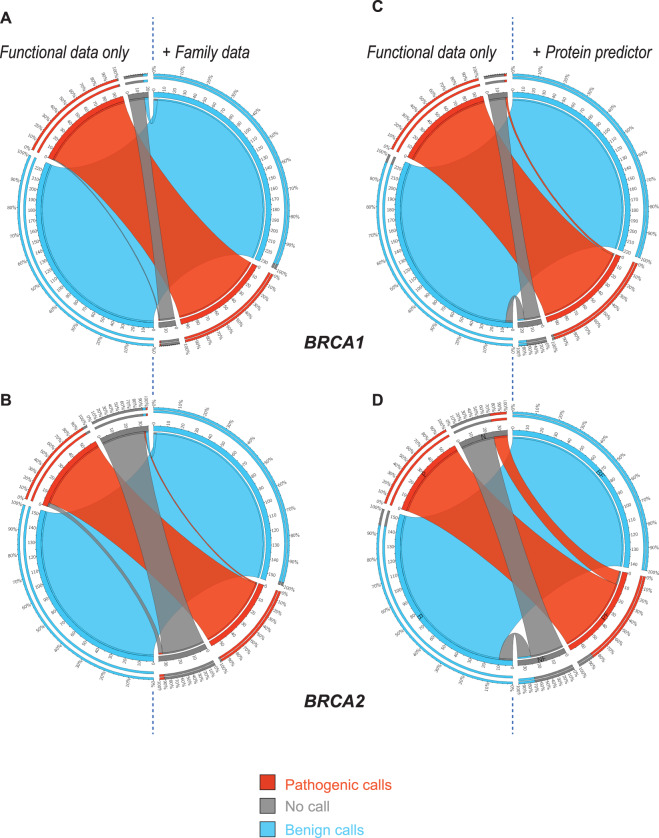
Changes in variant calls upon addition of family or protein predictor data. Circos plots illustrating changes in pathogenic calls (red ribbons), benign calls (blue ribbons), and no-calls (gray ribbons) with functional data-only (left half of the plot) and with the addition of family (**A, B**) or protein predictor data (**C, D**) for BRCA1 (**A, C**) and for BRCA2 (**B, D**). Outermost rings in each segment on the left side (starting calls) of the plot depicts the percentage of variants in function-only calls that remained in the same category after addition of family or protein predictor data. Outermost rings in each segment on the right side (final calls) depicts the contribution of variants that started as calls in the same category.

The classification status of the majority of variants was unchanged with the addition of the family data. However, four *BRCA1* no-calls became benign and one pathogenic call became a no-call (Fig. 2A). All of these variants are currently VUS. Scatter plots of the log posterior odds of VUS pathogenicity using the functional data-only (*x*-axis) versus the combined functional and family data provide more resolution (Supplementary Fig. 2). There are six differences for *BRCA2*: two no-calls became benign and one became pathogenic and three pathogenic calls became no-calls (Fig. 2B). Four of these are VUS, two are known pathogenic variants. A comparable number of *BRCA1* (*n* = 5) and *BRCA2* variants (*n* = 6) shift into or out of a no-call region (Fig. 2A, B). Adjusted for the ‘size’ of the no-call region (there are about twice as many *BRCA2* variants in the function-only model no-call region (*n* = 35) than in the corresponding *BRCA1* region (*n* = 19)), the fraction of *BRCA1* variants to shift classifications is larger than for *BRCA2* (but is still small as a fraction of the total number of variants). No variants changed from pathogenic to benign or benign to pathogenic (Fig. 2A, B). Most of the changes observed were cases very near a classification threshold (these regions are highlighted in Supplementary Fig. 2). Among variants predicted by the original VarCall to be benign, 74% (37/50) of *BRCA1* and 97% (59/61) of *BRCA2* variants had decreased odds of pathogenicity with the addition of the family data. Likewise, among variants predicted to be pathogenic, 22% (7/32) of *BRCA1* and 74% (26/35) *BRCA2* variants had increased odds of pathogenicity. These patterns are reflected in the plotted loess regression curves in Supplementary Fig. 2.

### Contribution of protein predictor data

While the classification status of the majority of variants remained unchanged with the addition of the protein predictor data as with the family data, differences were more frequent and pronounced, especially for *BRCA2* (Fig. 2C, D). Among *BRCA1* variants seven benign calls including a known benign variant became no-call VUS, and two no-call VUS became pathogenic (Fig. 2C). Similarly, among *BRCA2* variants ten benign calls including a known benign variant became no-call VUS, and ten no-call VUS became pathogenic (Fig. 2D). While the number of *BRCA2* variants to shift into or out of the no-call region is larger than for *BRCA1*, as a fraction of the variants in the gene’s function-only no-call region these values are comparable. The shift in classification categories for *BRCA2* variants reflected an overall increase in the posterior log-odds of pathogenicity that resulted from the addition of the protein predictor data, which strengthened the original VarCall estimates for likely benign *BRCA1* VUS, but had little effect on the variants called as likely pathogenic. The log-odds of pathogenicity of 150 of 229 variants called likely benign by the original function-only model decreased with the addition of the protein predictor data, while only 37 of 99 variants called likely pathogenic experienced an increase. In contrast, *BRCA2* variants of all classifications experienced an increase in the log-odds of pathogenicity, with 146 of 154 variants called likely benign and 56 of 59 variants called likely pathogenic by the original VarCall displaying increased odds of pathogenicity with the addition of the protein predictor data.

**Fig. 3:**
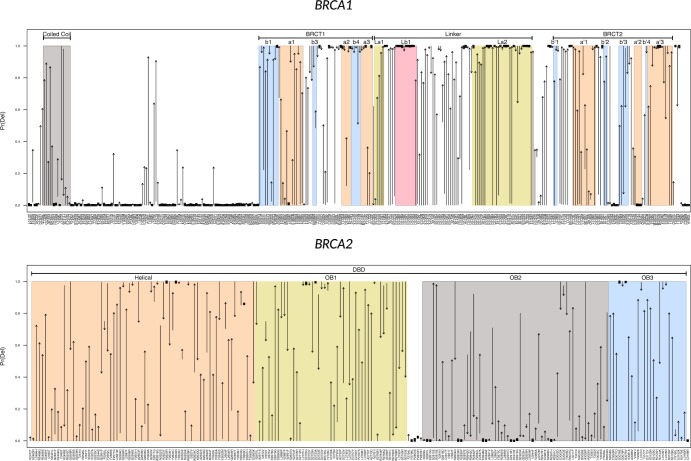
Differences in estimated probabilities of pathogenicity for *BRCA1* (top) and *BRCA2* (bottom) based on the protein predictor data alone and the functional assay data alone. The data for each variant are depicted as an arrow from the estimated probability given only the functional assay data to the estimated probability given only the protein predictor summary data. Variants are plotted on the *x*-axis in order of the location of the altered residue. Functional regions are highlighted and labeled.

Differences in probabilities of pathogenicity estimated using only the functional assay data and those estimated using only the protein predictor data are shown in Fig. 3. The probabilities of 42% of *BRCA1* VUS changed by more than 0.1, 27% changed by more than 0.5, and 13% by more than 0.9. These figures were 63%, 29%, and 4%, respectively, for *BRCA2*. The pathogenicity probabilities estimated using only the in silico data were higher, on average than those estimated using only the functional assay data for both *BRCA1* (mean difference = 0.22; sd = 0.40) and *BRCA2* (mean difference = 0.13; sd = 0.41). In *BRCA1*, the mean difference was 0.22 (SE = 0.050) in the BRCT1 domain, 0.28 (SE = 0.049) in the BRCT2 domain, 0.13 (SE = 0.10) in the Coiled Coil domain, 0.42 (SE = 0.043) in the Linker region and 0.073 (SE = 0.034) in the remaining, unannotated regions. In *BRCA2*, the mean difference was 0.17 (SE = 0.048) in the Helical region of the DNA Binding Domain, 0.087 (SE = 0.059) in OB1, 0.052 (SE = 0.053) in OB2, 0.34 (SE = 0.082) in OB3 and 0.015 (SE = 0.16) in the unlabeled regions. These results, coupled with the poor operating characteristics of the protein predictor model (Table 1), suggest that the discrepancies are driven by a lack of calibration and refinement in the protein predictors.

The effects of the Align-GVGD score were similar to those associated with the in silico protein predictor data. The effects on *BRCA1* classifications were modest. Sensitivity and specificity estimates were unchanged (Table 1) with only eight benign calls changing to no-call VUS and four changing from no-call VUS to pathogenic. Differences in log-odds of pathogenicity (see Supplementary Fig. 2, left panel) were small. The effects of adding Align-GVGD scores on *BRCA2* classifications were more striking, with 44 variants called benign from functional data alone being reclassified as no-call VUS. Furthermore, 23 variants classified as no-call VUS reclassified as pathogenic. As a result, the estimated sensitivity decreased, the estimated specificity increased and differences in log-odds of pathogenicity were large (Supplementary Fig. 3). The systematic shift evident in this plot can be traced to a discrepancy between the Align-GVGD scores and the variant-level probabilities based on the functional data alone: across all variants, the Align-GVGD probabilities were larger by 0.155 (95% CI 0.099 to 0.212; *p* = 1.30 × 10^−7^); among the VUS by 0.179 (95% CI 0.116 to 0.242; *p* = 6.64 × 10^−8^) and, in contrast, among *BRCA1* VUS by −0.00272 (95% CI −0.0465 to 0.0410; *p* = 0.903).

The Align-GVGD score also strengthened the original VarCall estimates for likely benign *BRCA1* VUS, but had little effect on the likely pathogenic variants or on *BRCA2* variants. The results we observe here may overstate Align-GVGD’s potential to improve predictive accuracy since it was also used as a prior probability in the multifactorial model^3^ used to establish the IARC classifications of the variants we use to assess accuracy.

## Discussion

We have extended the VarCall model to incorporate additional forms of data related to the pathogenicity of VUS and have characterized the predictive performance based on two functional data-only and 13 unique combinations of functional assay data, in silico protein predictions, Align-GVGD score, and a summary of cancer family history. The resulting model, referred to as VarCall XT, is structured to facilitate the addition of further forms of evidence, including data from additional functional assays, genetic analyses, and tumor pathology characteristics. VarCall XT provides a prediction of the probability that each VUS is pathogenic based on data from multiple sources, thus providing a measure for assessing the strength of evidence in favor of or against pathogenicity and for inclusion in VUS classification schema.

Available data for variant classification are expected to become richer over time as more individuals are tested, new models for in silico prediction of protein function are developed, other functional assays are developed and standardized, assays are extended to more VUS, and other forms of evidence become available. VarCall XT provides a flexible platform for updating predictions of pathogenicity as these new data arise. VarCall XT assumes that VUS have binary risk profiles, behaving like the wild-type sequence or pathogenic, protein-truncating variants. However, there are likely to be hypomorphic variants and distribution of penetrance values intermediate to these two extremes. The VarCall model can be easily adapted to allow for a different distribution of risk profiles.

As currently configured, VarCall identifies variants of known pathogenicity with high sensitivity and specificity with functional assays contributing the greatest predictive power to the model. Indeed, considering the functional data alone, 98.4% of *BRCA1* variants of known disposition were correctly called (one known benign was a no-call VUS) and 97% of *BRCA2* variants were correctly called (one known pathogenic variant was a no-call VUS). This level of accuracy is not surprising as these functional assays are well-calibrated^7,8^. While it is difficult to identify a single best model, the contribution of the functional data is clear. The least accurate model that included the functional data was (much) more accurate than the most accurate model that did not include functional data. This can be explained, at least in part, as an artifact of statistical power: the variants that we focus on each gene have multiple measurements made by the functional assays. The available family data is much weaker: because many variants are seen rarely, truly informative family data is the exception, not the rule. The utility of in silico protein predictors and Align-GVGD score for the prediction of individual VUS is far less clear. Coverage of the protein predictor data is complete, but its ability to separate pathogenic from neutral *BRCA1* and *BRCA2* variants is imperfect, this may be because the protein predictor models have been developed to identify broader classes of likely functional variation.

Further, the family data corroborated the functional assays despite their limited extent and the lack of co-segregation data. With continued growth in testing, there is the potential for family history summaries to become much more informative, but this outcome will depend on the availability and quality of these data. Our results highlight the fact that, despite the current shortcomings of these sources of data, accurate classification of VUS can rely heavily on functional assay data. This is and will continue to be especially important for rare VUS that lack informative family data.

Importantly, the functional assays described above cannot be used to classify splice variants. Thus, specific RNA-based splicing assays or functional assays that allow for endogenous splicing within *BRCA1* and *BRCA2* may be needed for the classification of potential splice variants. Importantly, splicing data can be incorporated into the VarCall XT model as they become available.

An inherent limitation in the VarCall XT approach is the historical definition of pathogenic missense variants in the *BRCA1* and *BRCA2* genes. Both the *BRCA1* transcriptional assay and the *BRCA2* homologous recombination assay were clinically calibrated using controls with properties that were well established in the literature. It is therefore not surprising that the most informative predictive model contains functional data, and hence the lowest Brier scores since that score reflects the *calibratedness* of the predictor. Family history and protein prediction scores contribute some amount of measurement error and bias to classification schemes. However, these effects will diminish as testing-based family data increases in scope and coverage and as protein prediction models increase in accuracy.

DNA testing is a powerful tool used to tailor medical care based on individual cancer risks. However, DNA testing can produce inconclusive results when a VUS is identified. In this common scenario, healthcare providers might not have the information needed to recommend appropriate preventive and early detection steps, or certain therapeutic treatments, such as PARP inhibitors, and family members may not be referred for genetic testing or enhanced screening. Our results indicate that combining functional assay with other forms of data regarding *BRCA1* and *BRCA2* missense variants can overcome this limitation and lead to a more accurate determination of whether a variant results in increased cancer risk.

## Methods

### *BRCA1* transcriptional assay (TA) data

The *BRCA1* transcriptional assay dataset has been described in detail elsewhere^9^. Briefly, the assay is based on the ability of the BRCA1 C–terminal region to activate the transcription of a reporter gene when fused to a heterologous transcription factor DNA binding domain (GAL4 or LexA)^10^. This validated assay has been shown previously to have high predictive sensitivity and specificity^7^.

In this dataset, 281 *BRCA1* VUS, 42 IARC class 1 or 2 (benign and likely benign by the IARC sequence variant classification scheme^11^) and 21 IARC class 4 or 5 variants (likely pathogenic and pathogenic), and two controls were assayed for transcriptional activity in 126 experimental batches (3,453 individual ratio measurements). Each batch included the wild-type (WT) as a positive control and p.(Met1775Arg) (IARC Class 5) as a negative control. Typically, three replicates of each variant were made within each batch. As previously^9,12^, we averaged the within-batch replicate log-ratio values and analyzed the resulting variant- and batch-level mean values.

### *BRCA2* homologous recombination (HR) assay data

The *BRCA2* homologous recombination assay and the associated experimental protocol have been described previously^8^. The *BRCA2* HR dataset analyzed here comprised 2233 individual log-ratio measurements made on a total of 248 variants. Of these, 22 were IARC class 1 or 2 variants that, together with the wild-type (WT) control, were labeled as benign in the analysis. In addition to the loss-of-function (LOF) control p.(Asp2723His), 11 IARC class 4 and 5 likely pathogenic and pathogenic variants were labeled as damaging to protein function. The remaining 168 variants were VUS and were unlabeled in the analysis. The data were collected in 162 experimental batches. The WT and LOF variants were replicated twice in every batch, while all other variants were replicated at least twice in at least one batch. As with the *BRCA1* assay data, we averaged the within-batch replicate log-ratio values for each variant and analyzed the 984 resulting within-batch means.

### In silico protein prediction data

In silico predictions from 27 protein prediction models for all assayed variants in *BRCA1* and *BRCA2* were obtained from the dbNSFP^13^ (v4.0b2) database using the BioR toolkit^14^. For simplicity, the predictions from each model were expressed as rank scores and scaled to a range between zero and one. Hence a score of 0.9 implied that the variant was more likely to be damaging than 90% of all other variants predicted by that method^15^. Because these metrics are correlated, we carried out a principal components analysis to identify the ten orthogonal linear combinations (PCs) summarizing a majority (90%) of variation in the metrics. Additional detail is available in the Supplement.

In addition, we separately investigated Align-GVGD^16^ as a predictor of variant pathogenicity. Tavtigian et al.^16^ provided point and 95% interval estimates of the probability that a given missense substitution in *BRCA1* or *BRCA2* was pathogenic based on a multiple sequence alignment ranging from humans to sea urchin.

### Family data

Using methods described elsewhere^17^, we estimated Bayes factors (BFs) in favor of variant pathogenicity based on family history summaries obtained from ~140,000 patients tested by Ambry Genetics for the presence of pathogenic variants in *BRCA1* and *BRCA2*. We included these quantities in our multifactorial models as described below.

Ambry Genetics declares that the Western Institutional Review Board has issued a regulatory opinion based on federal regulation 45 CFR 46 guidance that the research, which is based on de-identified data, does not involve human subjects. Therefore, informed consent was not required.

### *BRCA1* and *BRCA2* VarCall models and multifactorial extensions

VarCall^7–9,12,18^ models comprise a family of models for functional assay data, have been applied to several different functional assays for *BRCA1* and *BRCA2,* and share the following common structure. They are formulated as Bayesian hierarchical models constructed around a core random-effects model for the functional data. The random-effects model decomposes variation in the assay readout into three sources: that explained by the variant, that related to the experimental batch and background variation. Variant-specific random effects are assumed to be normally distributed with a mean and variance parameter that depends on whether the variant is damaging or not. This implies a two-component normal mixture model. Hence, while the first level of the model is a mixed-effects regression model, the second level is a Gaussian classification model. Classification models provide a more powerful alternative to approaches based on discriminant analysis and clustering, allowing for more accurate classifications with modest to small numbers of labeled samples^19–23^ (See Supplemental Methods for more detail).

The posterior probability that a given variant is pathogenic based on this classification model is the probability that the variant’s random effect parameter is drawn from the pathogenic component of the mixture model. To estimate this probability, we assign to each variant a binary variable, *D*, that indicates whether it is damaging (*D* = 1) or not (*D* = 0). The subset of variants assayed as positive or negative controls, i.e., those that are known or very likely pathogenic (Class 4/5) or known or very likely benign (IARC Class 1/2) have *D* set to 0 or 1 as appropriate, i.e., are “labeled,” while the values of *D* for the VUS are set to missing, i.e., are “unlabeled.” To avoid circularity, no functional data were used to establish the IARC classification of pathogenic and benign known variants. The IARC classification is based on the multifactorial model^2,3^ which does not include functional data. The Align-GVGD score was, however, used as a prior probability in the multifactorial model^3^ to obtain the IARC classification, raising the possibility that we may overestimate the accuracy of VarCall XT models that include Align-GVGD. To examine the sensitivity of our classifications to the fraction of labeled variants, we fit the *BRCA1* and *BRCA2* function-only VarCall models with only the WT and negative controls (p.(Met1775Arg) for *BRCA1* and p.(Asp2723His) for *BRCA2*) labeled for comparison. The formal description of VarCall and the extensions found in the supplement are based on those presented in refs. ^8,12,24^.

The multifactorial models investigated here extend the VarCall model and are distinguished by the variant-specific metrics (functional assay data, in silico protein predictor summaries, Align-GVGD score, family history summary). Future models that involve additional forms of evidence can be obtained by extending the model by following the approach described in the Supplement.

For each gene, we evaluated two progression series of multifactorial VarCall models: (1) beginning with the family data BFs and cumulatively adding the in silico sequence-based predictor data, the Align-GVGD score and, finally, the functional assay data in order to assess how functional data add to classification by family and in silico data; (2) beginning with the standard VarCall model for the functional assay data and cumulatively adding the family data, the in silico data and Align-GVGD score to assess whether adding family and in silico data improve classification based on functional data. Both series culminate in the full multifactorial model. The Align-GVGD in silico predictor was added separately because it is currently part of the established multifactorial model for BRCA1 and BRCA2 VUS classification^25^. To identify the most reliable model we evaluated the remaining models that include at least one of the four data types, for a total of 15 classification models.

### Model evaluation

For each gene, we compared each of the 15 VarCall models on basis of the classification probabilities assigned to the variants of known disposition in the context of a leave-one-variant-out analysis. We systematically left each unlabeled, treating it as if it were a VUS, refit the model, and recorded the variant’s estimated classification probability. Based on this value and thresholds established in the literature^9,11^, we classified the variant as ‘benign’ if Pr(*D* = 1 ∣ Data) < 0.05, as ‘pathogenic’ if Pr(*D* = 1 ∣ Data) ≥ 0.99 and left it unclassified otherwise; to distinguish these from the known classifications, we refer to these categories as ‘called benign’, ‘called pathogenic’ and ‘not called,’ respectively.

Using these estimated classifications, we compute and report model- and empirical, count-based estimates of the operating characteristics of the VarCall models. The empirical predictive summaries include tabulations of the estimated classifications conditional on known pathogenicity status; raw predictive accuracy rates that treat variants in the no-call category as incorrectly classified; and the scaled Brier score^26^, which summarizes the discordance between the binary classifications, *D*_*v*_, and the predicted probabilities, Pr(*D*_*v*_ ∣ Data) that result from a given model. We also compute Bayesian estimates of the sensitivity, specificity, false-positive, false-negative, and no-call rates associated with each VarCall model. In particular, for each model, we estimate the conditional distributions over the estimated classification categories given known disposition based on a Dirichlet-multinomial model for the observed counts assuming Jeffrey’s prior on the multinomial probability vector. We compute and report the posterior mean and 95% Bayesian high-density region (HDR) interval estimates for each operating characteristic based on the fitted model.

### Supplemental data

The supplemental data file includes a detailed description of the models; results of an analysis of sensitivity to the fraction of labeled variants; a table of empirical summaries of the 15 VarCall models for *BRCA1* and *BRCA2*; the protein predictor data; a figure of density plots of the top ten principal components of the protein predictor data; a figure of scatter plots of the log-odds of pathogenicity computed using the function data alone versus the function data combined with the Align-GVGD score; and a figure comparing classification probabilities when all known variants are labeled to those computed when only the control variants are labeled.

### Reporting summary

Further information on research design is available in the Nature Research Reporting Summary linked to this article.

## Supplementary information

Supplementary Material

Reporting Summary

Supplemental Data 1

## Data Availability

The data are available from the corresponding author upon request.

## Code availability

The analysis scripts are available from the corresponding author upon request.
