# Supplementary material for: An integrative model for the comprehensive classification of BRCA1 and BRCA2 variants of uncertain clinical significance
Source: NPJ Genom Med. 2022 Jun 3;7:35. doi: 10.1038/s41525-022-00302-3 (PMC9166814; doi:10.1038/s41525-022-00302-3)
Supplement: Supplementary file 1 — Supplementary Material [file 41525_2022_302_MOESM1_ESM.pdf]

Supplementary Material
A Multifactorial Model for the Classification of *BRCA1* and *BRCA2* VUS

Edwin S. Iversen, Jr.[*], Gary Lipton[*], Steven N. Hart[†], Kun Y. Lee[‡], Chunling Hu[‡], Eric C. Polley[‡], Tina Pesaran[§], Amal Yussuf[§], Holly LaDuca[§], Elizabeth Chao[§], Rachid Karam[§], David E. Goldgar[¶], Fergus J. Couch[‡,‖], and Alvaro N. Monteiro[**,‖]

April 29, 2022

[*]Department of Statistical Science, Duke University, Durham, NC 27708.
[†]Department of Health Sciences Research, Mayo Clinic, Rochester, MN 55901.
[‡]Department of Laboratory Medicine and Pathology, Mayo Clinic, Rochester, MN 55902.
[§]Ambry Genetics Corporation, Aliso Viejo, CA 92656.
[¶]Department of Dermatology, University of Utah School of Medicine, Salt Lake City, UT 84132.
[‖]Co–Senior Author
[**]Cancer Epidemiology Program, H. Lee Moffitt Cancer Center & Research Institute, Tampa, FL 33612.

Supplementary Methods

In Silico Protein Prediction Data

Because these metrics are correlated (49% of pairwise correlations exceeded 0.5 and these values ranged up to 0.96), the information on protein function was partially redundant. For this reason, we carried out a principal components analysis to identify the orthogonal linear combinations (PCs) summarizing a majority (90%) of variation in the metrics using the combined data for both genes; the first ten PCs were sufficient for this purpose. We examined the empirical distributions of the PCs as functions of gene and a classification of the variant based on the gene–specific VarCall models fit using only the functional assay data (these are described below). These distributions could not easily be modeled by standard parametric distributions (see Supplementary Figure 1). Therefore, each PC was transformed to a twenty level ordinal factor based on quantiles. We used these factors to summarize the *in silico* protein predictor data in the subset of multifactorial VarCall models described below that include these data.

Family Data

Using methods described elsewhere [1], we estimated Bayes factors (BFs) in favor of variant pathogenicity based on family history summaries obtained from approximately 140,000 patients tested by Ambry Genetics for the presence of pathogenic variants in *BRCA1* and *BRCA2*. Briefly, we developed *BRCA1*– and *BRCA2*–specific logistic regression models to predict the presence or absence of a known pathogenic variant in the gene given a set of features of personal and family history of cancer abstracted from patient surveys. Features included personal and family history of breast and ovarian cancer, age at cancer onset and presence of pancreatic and/or prostate cancer. When constructing these models, we excluded data from patients testing positive for a *BRCA1* or *BRCA2* VUS as well as those with a known pathogenic variant in another breast/ovarian cancer disease gene.

We then employed these models (to the Ambry VUS data) to predict posterior mutation probabilities, p_v, conditional on the observed family data for each VUS, v, included in our functional

assays that was observed in one or more patient tested by Ambry Genetics. We estimated the prior (to the Ambry VUS data) probability of pathogenicity, p_0 by the prevalence of known pathogenic variants identified in the gene by the testing. The Bayes factor is the ratio of the posterior odds of pathogenicity to the prior odds: $\mathrm{BF}_v = p_v(1-p_0)/((1-p_v)p_0)$. We included these quantities in our multifactorial models as described below.

BRCA1 and *BRCA2* VarCall Models and Multifactorial Extensions

VarCall Embeds a Gaussian Classification Model

VarCall [2] models are formulated as Bayesian hierarchical models constructed around a core random effects model for the functional data in which the first level of the model is a mixed effects regression model and the second level is a Gaussian classification model.

Model–based clustering, classification and discriminant analysis are distinguished from one another based on the fraction of objects (0%, between 0% and 100% and 100%, respectively) that have a known component membership (label) attached when estimating the parameters of the model [3]. Since unlabeled samples provide information about the mixture component parameters, in some cases approaching that of labeled samples [4], it is possible to informatively classify data even when no samples have a known classification [3, 5]. However, the statistical efficiency for sample classification increases as the fraction of labeled objects increases and with the degree of separation between the two components of the model [6, 7].

The posterior probability that a given variant is pathogenic based on this classification model is the probability that the variant's random effect parameter is drawn from the pathogenic component of the mixture model. As a device to estimate this probability, we assign to each variant a binary variable, D, that indicates whether it is damaging ($D = 1$) or not ($D = 0$). The subset of variants assayed as positive or negative controls, i.e. those that are known or very likely pathogenic (Class 4/5) or known or very likely benign (IARC Class 1/2) have D set to 0 or 1 as appropriate, i.e. are "labeled," while the values of D for the VUS are set to missing, i.e. are "unlabeled." The formal description of VarCall and its extensions is based on those presented in Zhou *et al.* (2005) [8], Iversen *et al.* (2011) [2] and Guidugli *et al.* (2018) [9].

Extending the VarCall Model

The family of VarCall models we describe herein share a common structure. All include variant–specific functional assay data (f_v, where v denotes the variant), while the multifactorial extensions add to this one or more of: the *in silico* protein predictor data (s_v), the Align–GVGD score (a_v) and the family history Bayes Factors (BFs) (h_v). We treat the functional, *in silico* and Align–GVGD data, when included, as response variables and the family history variable, when included, as a predictor variable. We elaborate on these choices below. We also condition on the design variable B_v indicating the experimental batch associated with each of the functional assay measurements on variant v. We treat the variant's classification, D_v, as a response variable when it is observed and marginalize over it when it is not (third term in Equation 1). Denoting by θ the model parameters, by Y_v the outcome measures, by Z_v the predictor variables and by X_v the design variables, the sampling model for the data is

$$\begin{aligned}\prod_{\{v\,:\,D_v \text{ is Known}\}} \Pr(Y_v, D_v \mid Z_v, X_v, \theta) \prod_{\{v\,:\,D_v \text{ is Unknown}\}} \Pr(Y_v \mid Z_v, X_v, \theta) \;=\; & \\ \prod_{\{v\,:\,D_v=0\}} \Pr(Y_v \mid D_v = 0, X_v, \theta) \Pr(D_v = 0 \mid Z_v, \theta) \quad & \times \\ \prod_{\{v\,:\,D_v=1\}} \Pr(Y_v \mid D_v = 1, X_v, \theta) \Pr(D_v = 1 \mid Z_v, \theta) \quad & \times \\ \prod_{\{v\,:\,D_v \text{ is Unknown}\}} \Big[\sum_{d=0}^{d=1} \Pr(Y_v \mid D_v = d, X_v, \theta) \Pr(D_v = d \mid Z_v, \theta)\Big]. & \end{aligned} \tag{1}$$

The sampling model includes two distinct factors: the first, $\Pr(Y_v \mid D_v, X_v, \theta)$, is a model for the outcome metric(s) conditional on variant classifications and (relevant) predictor and design variables; the second, $\Pr(D_v \mid Z_v, \theta)$, specifying the prior probability that a variant is damaging conditional on the relevant predictor and design factors. In the above, we assume that D_v is conditionally independent of X_v given Z_v and θ (i.e. we assume that batching in the assay experiments is not predictive of variant pathogenicity status) and that Y_v is conditionally independent of Z_v given D_v, X_v and θ (i.e. we assume that correlations between the family data, X_v, and the components of Y_v

are explained entirely by pathogenicity status, D_v). The various multifactorial VarCall models are distinguished by the measures included in Y_v and Z_v and the (co)variables included in X_v.

Each type of data could have been included either through the conditional (prior/prospective) model for the indicator D or as a retrospective component conditional on D. Given that the family data LR's are proportional to posterior odds, we felt it would be most natural to include these data as a prior as described below. In addition, it was also not clear how to formulate a good and parsimonious retrospective model for these data. Align–GVGD was developed for use as a prior and it would have been natural to use it in that role here (as we did in Guidugli *et al.* (2018) [9]), but instead we decided to include it, as well as the protein predictor data, in the retrospective component. In this way, the correlations between these variables and pathogenicity status are each modeled via components structured as Pr(X|D) instead of Pr(D|X). The predictive power of each data type should be comparable when carefully modeled either way, but the retrospective approach has been shown to be be more statistically efficient than the prospective approach in a setting similar to the current one [10]. This was an important consideration influencing our decisions.

The VarCall Model for Functional Assay Data

In the standard VarCall model described in Guidugli *et al.* (2018) [9], Z_v is not included as there are no predictor variables, $Y_v = f_v$ and $X_v = B_v$, where f_v represents the set of batch–level assay measurements for variant v and B_v identifies the batch for each. We index experimental batches using b and denote by $\bar{f}_{vb}$ the average of the replicate log–ratio values for variant v from batch b,

The VarCall model for the functional assay data assumes that the log–ratio measurements are conditionally independent given batch, variant and pathogenicity status, so that

$$\Pr(Y_{v\prime} \,|\, D_{v\prime}, X_{v\prime}, \theta) \;=\; \Pr(f_{v\prime} \,|\, D_{v\prime}, B_{v\prime}, \theta) \;=\; \prod_{\{(v,b)\,:\,v=v'\}} \Pr(\bar{f}_{vb} \,|\, D_v, b, \theta). \tag{2}$$

The model, $\Pr(\bar{f}_{vb} \,|\, D_v, b, \theta)$, for the batch–level log–ratio data, $\bar{f}_{vb}$, is a Bayesian random effects regression model in which there are batch level (denoted β_b), batch scale (denoted τ_b) and variant–specific (denoted η_v) random effects and a batch–specific error variance. This is motivated by the batch–to–batch variation in both the magnitude (location) and range (scale) of the log–ratio

measurements such that

$$\frac{(\bar{f}_{vb} \; - \; \beta_b)}{\tau_b} \; = \; \eta_v + e'_v$$

where $e'_v \sim \mathrm{N}(0, \psi^2)$ and, therefore, that

$$\bar{f}_{vb} \; = \; \beta_b \; + \; \tau_b \eta_v + e_v$$

where $e_v \; \sim \; \mathrm{N}(0, \tau_b^2 \psi^2)$. We include two constraints to the random effects model to make these effects identifiable, one to constrain additive shifts of the form $(\beta, \; \tau\eta) \; \rightarrow \; (\beta + c, \; \tau\eta - c)$ and one to constrain arbitrary multiplicative scaling of the form $(\tau, \; \eta) \; \rightarrow \; (\tau/c, \; c\eta)$. These constraints are effected by specifying the wild type variant and the loss–of–function control effects, denoted η_{WT} and η_{LOF}, respectively.

The three sets of random effects are assumed to be mutually independent and normally distributed. Parameters of the batch–specific random effects distributions in the *BRCA1* model are indexed by the amino acid context employed in the batch. Hence, the variant–specific random effects distribution is a two component normal mixture model with component membership determined by D_v and with component–specific mean and variance terms. This, the core of the VarCall model, coupled with the prior probability of component membership, specifies a Gaussian classification model for variant pathogenicity given the functional assay data.

Multifactorial Extension of the VarCall Model

The multifactorial models that we investigated here are distinguished by the variant–specific metrics included in Y_v and Z_v in Equation 1. In addition to f_v, Y_v may include one or both of the *in silico* protein predictor summaries s_v and the Align–GVGD score a_v and Z_v may include the case control family history summary h_v or be left out of the model. Clearly there is room to structure the model differently, for example by including one or both of s_v and a_v in Z_v instead of Y_v; the choices we made here were based on considerations including model flexibility and convenience as well as statistical efficiency. Future models that involve additional forms evidence can be obtained by adding the appropriate variables to X_v, Y_v and Z_v in Equation 1 and extending the model, in the

spirit of the description below, to accommodate each.

In the model including all the forms of evidence, we assume that $\Pr(D_v \mid Z_v, \theta) = \Pr(D_v \mid h_v, \theta)$ and that

$$\Pr(f_v, s_v, a_v \mid D_v, B_v, \theta) = \Pr(f_v \mid D_v, B_v, \theta) \Pr(s_v \mid D_v, \theta) \Pr(a_v \mid D_v, \theta), \tag{3}$$

i.e. that the forms of evidence f_v, s_v and a_v are conditionally independent given a variant's pathogenicity status and the model's parameters, and that only the functional assay data depends on the batching variable. Models that include only one or the other of s_v and a_v are obtained by removing the component associated with the other from Equation 3; models that do not include h_v are obtained by simplifying $\Pr(D_v \mid h_v, \theta)$ to $\Pr(D_v \mid \theta)$ as described below.

We also construct and evaluate a nested progression of models that exclude the functional assay data. These allow us to quantify the predictive accuracy of combinations of the *in silico* and family data alone, prior to the introduction of the functional assay data. These models are constructed by omitting the term for f_v in Equation 3 and including the remaining relevant terms as described above.

The Model $\Pr(D_v \mid h_v, \theta)$ for Variant Pathogenicity Given the Family History Data

We parameterize the model of variant pathogenicity status given the family history data as a logistic regression on the log of the family history Bayes factor, BF_v, placing a diffuse (SD=10) mean zero normal prior distribution on the intercept term and an independent normal mean one, standard deviation two prior distribution on the coefficient of the log BF. The rationale behind the choice of prior mean values is that, when the parameters equal these, the model is equivalent to setting the probability of pathogenicity equal to the (posterior) probability of variant pathogenicity given the family history summaries, i.e. p_v, when the prior probability of variant pathogenicity is taken to be 0.5. However, the prior variances allow room for the parameter values to depart significantly from these values. We set $\mathrm{BF}_v = 1$ for variants that did not appear in the Ambry testing data set, corresponding to the case where $p_v = p_0$, i.e. the probability of variant pathogenicity posterior to the analysis of the Ambry family data is equal to its prior value.

The Model $\Pr(s_v \mid D_v, \theta)$ for the *In Silico* Data

Our prior experience [11] developing models to predict the functional status of VUS in *BRCA1* and *BRCA2* given data from an ensemble of *in silico* protein predictor models suggests that the operating characteristics of these models, as measured against classifications derived from the functional data, vary from gene to gene. To this end, we introduce a variant–specific binary indicator, S_v, for whether the variant is detrimental to function (=1) or not (=0) given the *in silico* data and model $\Pr(S_v \mid D_v, \theta)$ as a Bernoulli trial with success probability π_1 when $D_v = 1$ and $(1-\pi_0)$ when $D_v = 0$. The parameters π_1 and π_0 can, therefore, be interpreted as the sensitivity and specificity, respectively, of functional classifications based on the protein predictor data when those based on the functional data are treated as the 'gold standard.'

We summarized the predictions of the 27 *in silico* protein predictor models using the top 10 PCs from a principal components analysis of the combined set of functionally assayed *BRCA1* and *BRCA2* variants, then transformed each into a twenty level ordinal factor. We denote by s_{iv} the ith ordinal factor and model the ten as conditionally independent of one another given the classification S_v. This leads to the following expression for the *in silico* factor that appears in multifactorial VarCall models:

$$\Pr(s_v \mid D_v, \theta) \;=\; \sum_{j=0}^{1} \Big[\prod_{i=1}^{10} \Pr(s_{iv} \mid S_v = j, \theta)\Big] \, \Pr(S_v = j \mid D_v, \theta).$$

Each of the ordinal PC–based factors is modeled as following a Dirichlet–multinomial model conditional on S_v. The Dirichlet cell probabilities are modeled as following a gamma distributed random walk, prior to the constraint that they sum to one, as a device to encourage smooth variation from one ordinally indexed level to the next.

The Model $\Pr(a_v \mid D_v, \theta)$ for the Align–GVGD Score

The Align–GVGD risk score was designed to function as a (prospective) probability of pathogenicity conditional on the variant assigned risk score. However, when it appears in a VarCall sampling model, it is as a retrospective term specifying the probability of the risk score conditional on

pathogenicity status. This parallels the treatment of the *in silico* protein predictor data and, allows estimation of sensitivity and specificity of Align–GVGD based classifications, here denoted A_v, relative to the gold standard D_v based on the functional assay data. We approach this as above, writing

$$\Pr(a_v \mid D_v, \theta) \;=\; \sum_{j=0}^{1} \Pr(a_v \mid A_v = j, \theta)\,\Pr(A_v = j \mid D_v, \theta).$$

We derived an expression for $\Pr(a_v \mid A_v, \theta)$ by multiplying Align–GVGD's conditional probabilities of pathogenicity given risk category, $\Pr(A_v \mid a_v, \theta) \;=\; \alpha_v$, by the marginal probabilities of the risk categories, $\Pr(a_v \mid \theta) \;=\; \pi_v$, to obtain a joint distribution, then computed the requisite probabilities via conditioning on A_v: $\Pr(a_v \mid A_v \;=\; 0, \theta) \;\;\propto\;\; \pi_v\,(1 - \alpha_v)$ and $\Pr(a_v \mid A_v \;=\; 1, \theta) \;\;\propto\;\; \pi_v\,\alpha_v$. For each risk category v, we modeled the associated α_v as beta distributed with parameters chosen so that the mean and 95% equal–tailed interval bounds of the distribution matched the estimates published by Tavtigian *et al.* [12], and modeled the vector of marginal probabilities $(\pi_1, \ldots, \pi_4)$ as uniformly distributed on the simplex.

VarCall Model Inference

We fit all models using Markov chain Monte Carlo algorithms implemented in the JAGS language [13]; all other computations were carried out in the R programming language [14]. We ran each sampler for 50,000 burn–in iterations followed by another 500,000 iterations. We ignored the burn–in iterations and retained every 25th of the subsequent samples for inference. We estimated marginal posterior means, standard deviations and intervals of the model parameters by sample–based counterparts and computed "Rao–Blackwellized" estimates [15] of the posterior probabilities $\Pr(D_v = 1 \mid \text{Data})$. We evaluated the top level distributional assumptions of the model by assessing the normality of estimated posterior expected standardized residuals, variant effects, batch location effects and logged batch scaling effects and, as in previous analyses [2, 9], we found no evidence of model misfit (data not shown).

Supplementary Results

Number of Labeled Variants

We examined sensitivity of our classifications to the fraction of labeled variants by fitting the *BRCA1* and *BRCA2* function–only VarCall models with only the positive (WT) and negative (M1775R for *BRCA1* and D2723H for *BRCA2*) controls labeled and comparing these results to those obtained when these as well as all variants of known status were labeled. The impact on predictions made by the *BRCA1* function–only VarCall model were negligible: no variants changed classification and the average absolute difference in predicted probabilities was 0.00091 (left panels of Supplementary Figure 4). The average absolute difference in probabilities was 0.0027 for *BRCA2* (right panels of Supplementary Figure 4). However five *BRCA2* variants changed classifications based on small changes in their estimated probabilities. All of these had estimated probabilities near a classification boundary (0.05 or 0.99; see the center right and lower right panels of the figure, respectively): E2847K changed from 0.047 (all known variants labeled) to 0.058 (only positive controls labeled), G2584D changed from 0.049 to 0.057, G2596R changed from 0.990 to 0.987, Q2491P changed from 0.042 to 0.051 and W2626C, a known pathogenic variant, changed from 0.9919 to 0.9897.

This analysis provides a clear demonstration of the relative power of classification models over discriminant analysis: Here we show that the accuracy of the VarCall model is only mildly sensitive to a major decrease in the number of labeled variants. Indeed, with only one labeled pathogenic and one labeled benign variant, classifications are almost as accurate as when many more (63 for *BRCA1* and 33 for *BRCA2*) are available.

Supplementary Tables

BRCA1 Model	Benign Variants ($n = 42$) Call −	No Call	Call +	Pathogenic Variants ($n = 21$) Call −	No Call	Call +	Overall Accuracy (%)
Function Only (Original VarCall model)	41	1	0	0	0	21	98.4
Function + Prot Pred	40	2	0	0	0	21	96.8
Function + Family Data	41	1	0	0	0	21	98.4
Function + AGVGD	41	1	0	0	0	21	98.4
Function + Prot Pred + Family Data	40	2	0	0	0	21	96.8
Function + Prot Pred + AGVGD	38	4	0	0	0	21	93.7
Function + Family Data + AGVGD	41	1	0	0	0	21	98.4
Function + Prot Pred + Family Data + AGVGD	38	4	0	0	0	21	93.7
Prot Pred + Family Data + AGVGD	28	11	3	0	12	9	58.7
Prot Pred + AGVGD	27	12	3	0	12	9	57.1
Family Data + AGVGD	3	39	0	1	20	0	4.8
Prot Pred + Family Data	27	15	0	0	13	8	55.6
Prot Pred Data Only	26	15	1	0	13	8	54
Family Data Only	3	39	0	0	21	0	4.8
AGVGD Data Only	0	42	0	0	21	0	0

BRCA2 Model	Benign Variants ($n = 22$) Call −	No Call	Call +	Pathogenic Variants ($n = 11$) Call −	No Call	Call +	Overall Accuracy (%)
Function Only (Original VarCall model)	22	0	0	0	1	10	97
Function + Prot Pred	21	1	0	0	0	11	97
Function + Family Data	22	0	0	0	3	8	90.9
Function + AGVGD	20	2	0	0	0	11	93.9
Function + Prot Pred + Family Data	21	1	0	0	1	10	93.9
Function + Prot Pred + AGVGD	18	4	0	0	0	11	87.9
Function + Family Data + AGVGD	20	2	0	0	0	11	93.9
Function + Prot Pred + Family Data + AGVGD	19	3	0	0	0	11	90.9
Prot Pred + Family Data + AGVGD	11	11	0	0	9	2	39.4
Prot Pred + AGVGD	10	11	1	0	9	2	36.4
Family Data + AGVGD	1	21	0	0	11	0	3
Prot Pred + Family Data	9	13	0	0	10	1	30.3
Prot Pred Data Only	8	14	0	0	9	2	30.3
Family Data Only	0	22	0	0	11	0	0
AGVGD Data Only	0	22	0	0	11	0	0

Supplementary Table 1: Classification summaries of the *BRCA1* and *BRCA2* predictive models. Frequencies of positive calls, no calls and negative calls among the known benign variants (columns 3 through 5) and among the known pathogenic variants (columns 6 through 8). Overall accuracy (column 9) is defined as the percentage of correctly called variants; 'no calls' are considered to be incorrectly called.

Supplementary Figures

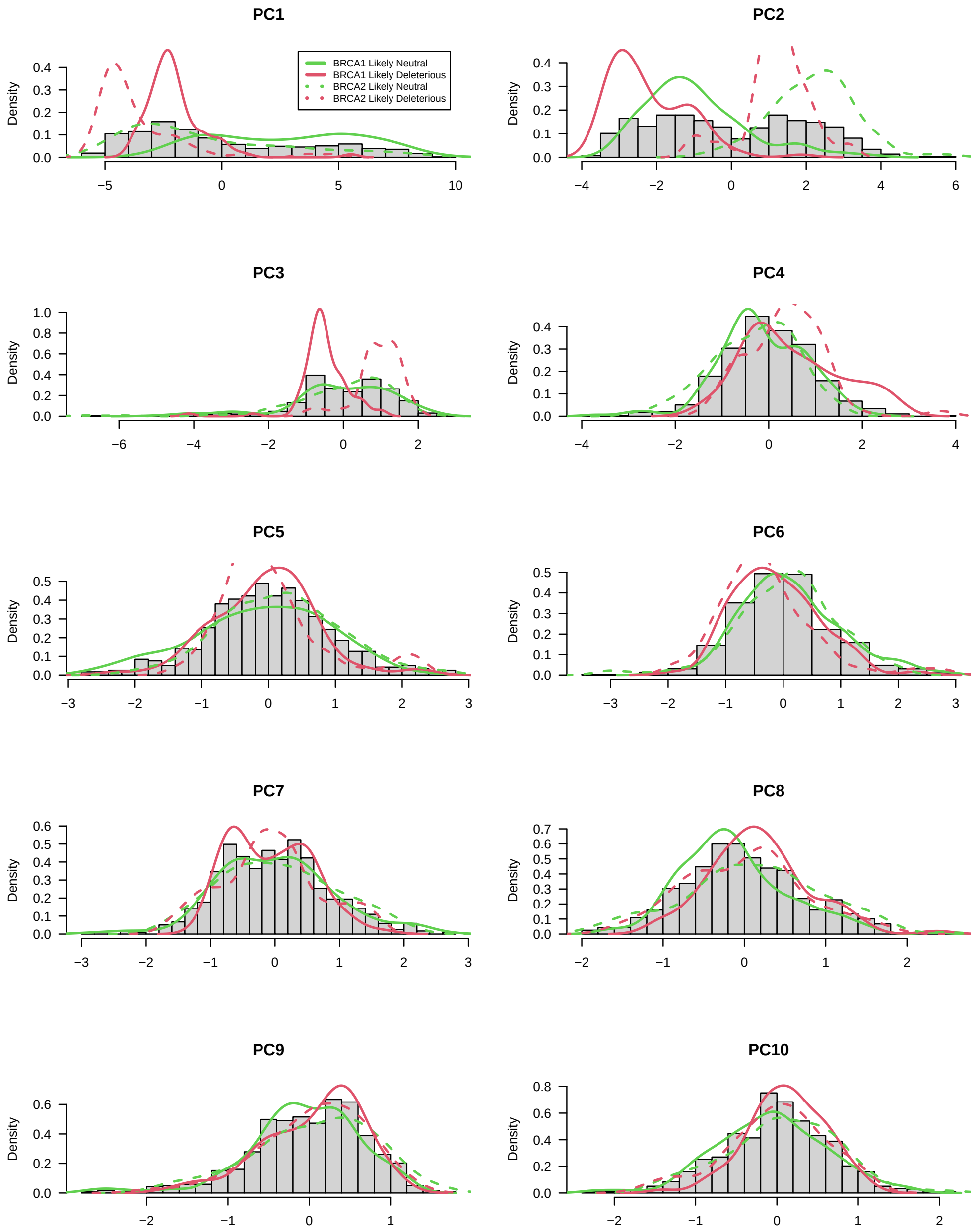

Supplementary Figure 1: Density estimates of the top ten principal components (PCs) of variation in the 27 *in silico* protein predictor variables. Histograms for each PC are overlayed with kernel density estimates of the distribution of the PC among likely benign *BRCA1* variants (solid green lines), likely benign *BRCA2* variants (dashed green lines), likely deleterious *BRCA1* variants (solid red lines) and likley deleterious *BRCA2* variants (dashed red lines). For purposes of this plot, variants were deemed likely benign (alt deleterious) if their posterior probability of being deleterious based on the gene–specific function–data–only model was less than 0.05 (alt greater than 0.99).

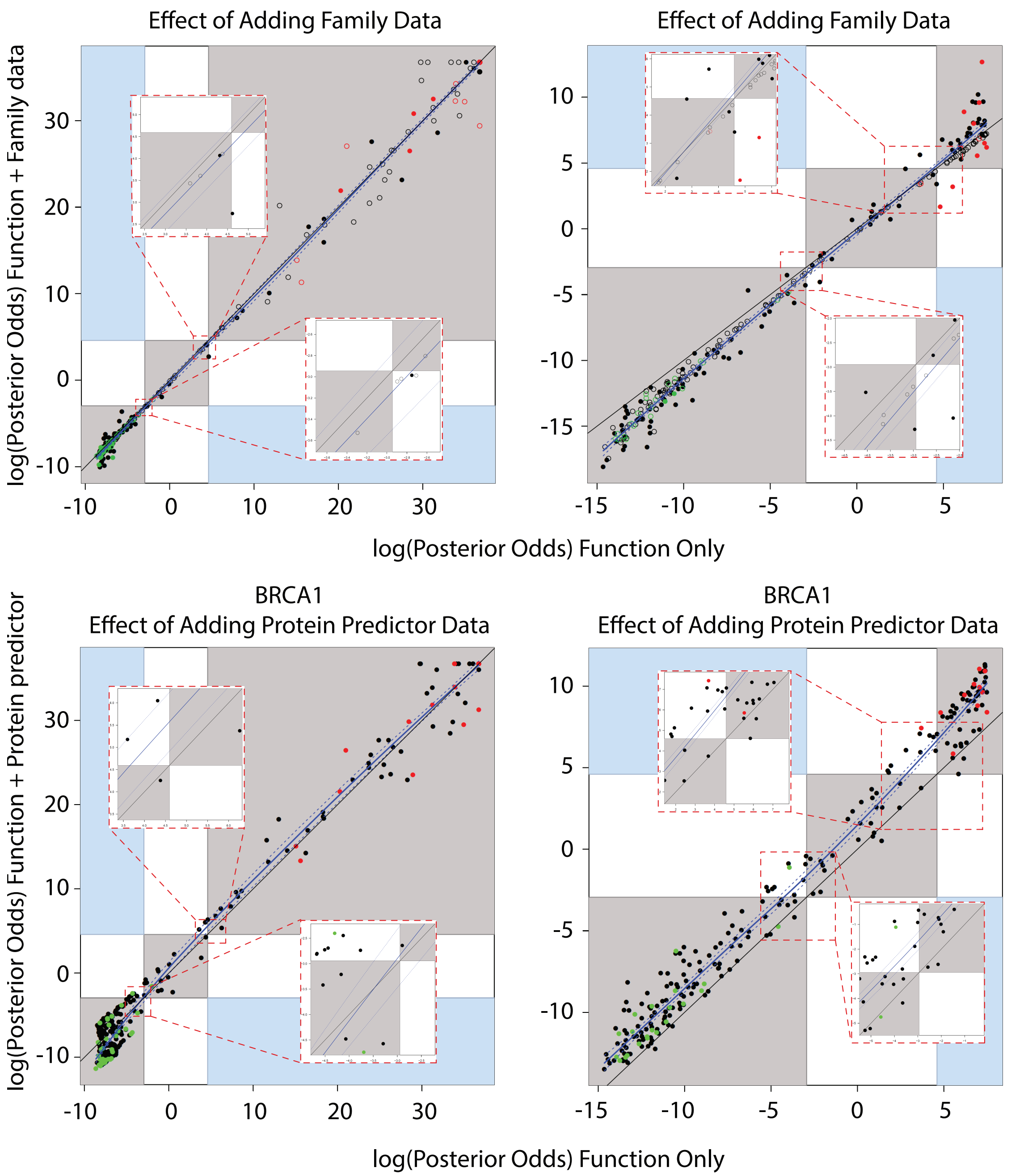

Supplementary Figure 2: Scatter plots, with loess smooths (blue lines; estimation SEs are dashed blue lines), of the logged posterior odds of VUS pathogenicity computed using the function data only (x–axis) against the same quantity computed combining the function with either the family data (top row) or the protein predictor data (bottom row) on the y–axis. VUS are plotted in black, benign variants in green and pathogenic variants in red. Variants with family data are plotted in the family data plots as solid circles and those without as hollow circles. Plots for the *BRCA1* models are in the left column and those for *BRCA2* are in the right column. Horizontal and vertical lines are drawn at the values of the log posterior odds corresponding to probabilities of 99% (∼ 4.6 on the log–odds scale) and 5% (∼ -2.9 on the log–odds scale). Variants that fall in a gray region do not change classification with the addition of the new form of data; those that fall in a white region change from a 'no call' to a pathogenic or benign call or the reverse. No variants are observed to change from a pathogenic to benign or benign to pathogenic call (blue regions). Insets show expanded views of the regions near classification boundaries.

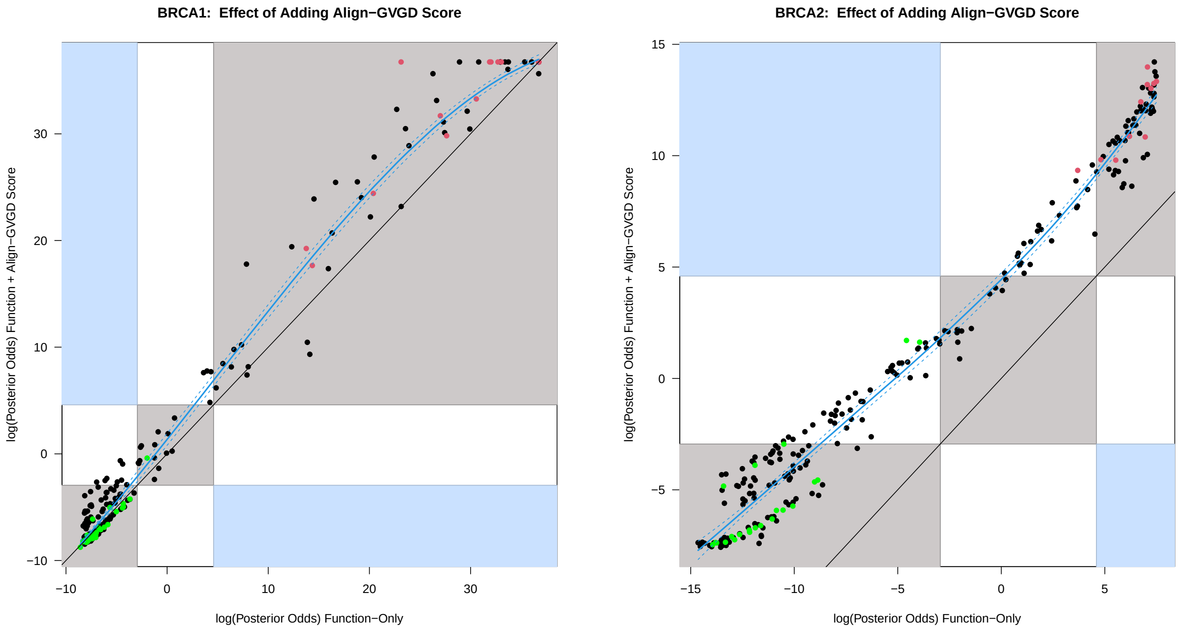

Supplementary Figure 3: Scatter plots of the logged posterior odds of VUS pathogenicity computed using the function data only (x–axis) against the same quantity computed combining the function data with the Align–GVGD score on the y–axis. VUS are plotted in black, benign variants in green and pathogenic variants in red. The *BRCA1* plot is in the left panel; the *BRCA2* plot is on the right. Horizontal and vertical lines are drawn at the values of the log posterior odds corresponding to probabilities of 99% (∼ 4.6 on the log–odds scale) and 5% (∼ -2.9 on the log–odds scale). Points that fall in a gray region correspond to variants that do not change classification with the addition of the form of data; points that fall in a white region correspond to variants that change from a 'no call' to a pathogenic or benign call or the reverse. No variants are observed to change from a pathogenic to benign or benign to pathogenic call (blue regions). loess smooths are plotted as blue lines; their estimation SEs are dashed.

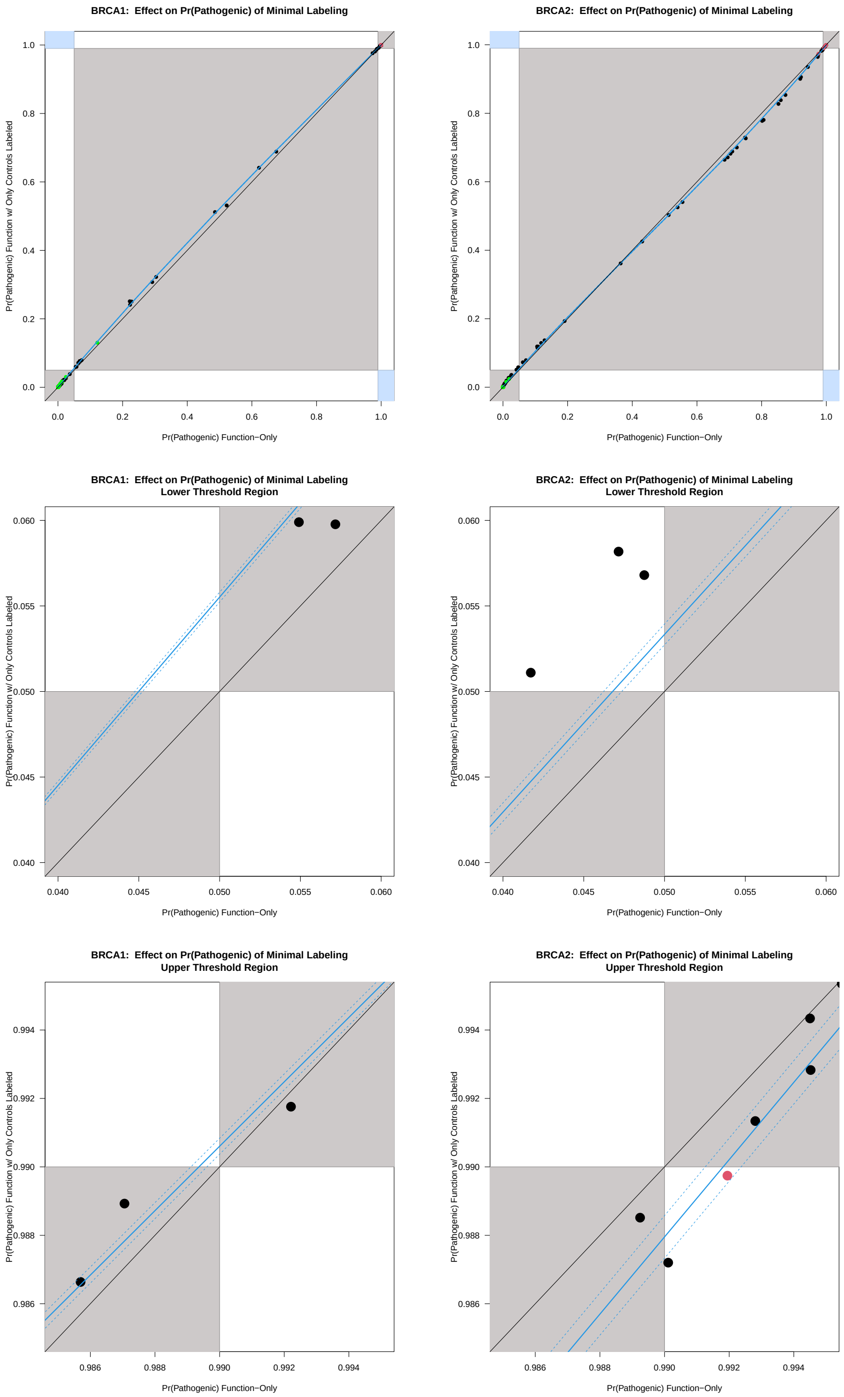

Supplementary Figure 4: Scatter plots of the posterior probabilities of VUS pathogenicity computed using the functional data only and with all variants of known disposition labeled (x–axis) against the same quantity computed when only the positive (WT) and negative (M1775R for *BRCA1* and D2723H for *BRCA2*) controls are labeled (y–axis). *BRCA1* plots in column 1, *BRCA2* in column 2; panels in row 2 focus in on the region of the lower classification threshold, while those in row 3 highlight the region around the upper threshold. Format is similar to that of Supplementary Figure 3.

References

[1] Li, H. *et al.* Classification of variants of uncertain significance in *BRCA1* and *BRCA2* using personal and family history of cancer from individuals in a large hereditary cancer multigene panel testing cohort. *Genetics in Medicine* **22**, 701–708 (2020).

[2] Iversen, E. S., Couch, F. J., Goldgar, D. E., Tavtigian, S. V. & Monteiro, A. N. A. A computational method to classify variants of uncertain significance using functional assay data with application to *BRCA1*. *Cancer Epidemiology, Biomarkers and Prevention* **20**, 1078–1088 (2011).

[3] McNicholas, P. D. *Mixture Model–Based Classification* (Taylor & Francis, Boca Raton, 2016).

[4] Dean, N., Murphy, T. B. & Downey, G. Using unlabelled data to update classification rules with applications to food authenticity studies. *Journal of the Royal Statistical Society, Series C (Applied Statistics)* **55**, 1–14 (2006).

[5] Fraley, C. & Raftery, A. E. Model–based clustering, discriminant analysis, and density estimation. *Journal of the American Statistical Association* **97**, 611–631 (2002).

[6] O'Neill, T. J. Normal discrimination with unclassified observations. *Journal of the American Statistical Association* **73**, 821–826 (1978).

[7] Ganesalingam, S. & McLachlan, G. J. The efficiency of a linear discriminant function based on unclassified initial samples. *Biometrika* **65**, 658—662 (1978).

[8] Zhou, Z., Iversen, E. S. & Parmigiani, G. Classification of missense mutations of disease genes. *Journal of the American Statistical Association* **100**, 51–60 (2005).

[9] Guidugli, L. *et al.* Assessment of the clinical relevance of *BRCA2* missense variants by functional and computational approaches. *American Journal of Human Genetics* **102**, 233–248 (2018).

[10] Efron, B. The efficiency of logistic regression compared to normal discriminant analysis. *Journal of the American Statistical Association* **70**, 892–898 (1975).

[11] Hart, S. N. *et al.* Comprehensive annotation of *BRCA1* and *BRCA2* missense variants by functionally validated sequence–based computational prediction models. *Genetics in Medicine* **21**, 71–80 (2018).

[12] Tavtigian, S. V., Byrnes, G. B., Goldgar, D. E. & Thomas, A. Classification of rare missense substitutions, using risk surfaces, with genetic– and molecular–epidemiology applications. *Human Mutation* **29**, 1342–54 (2008).

[13] Plummer, M. JAGS: A program for analysis of Bayesian graphical models using Gibbs sampling. In Hornik, K., Leisch, F. & Zeileis, A. (eds.) *Proceedings of the 3rd international workshop on distributed statistical computing (DSC 2003)* (ISSN 1609-395X, Vienna, Austria, 2003). URL `http://www.R-project.org/conferences/DSC-2003`.

[14] R Development Core Team. *R: A Language and Environment for Statistical Computing.* R Foundation for Statistical Computing, Vienna, Austria (2016). URL `http://www.R-project.org`. ISBN 3-900051-07-0.

[15] Gelfand, A. & Smith, A. F. M. Sampling–based approaches to calculating marginal densities. *Journal of the American Statistical Association* **85**, 398–409 (1990).
